# Feeding Our Immune System: Impact on Metabolism

**DOI:** 10.1155/2008/639803

**Published:** 2008-02-25

**Authors:** Isabelle Wolowczuk, Claudie Verwaerde, Odile Viltart, Anne Delanoye, Myriam Delacre, Bruno Pot, Corinne Grangette

**Affiliations:** ^1^Laboratoire de Neuro-Immuno-Endocrinologie, Institut Pasteur de Lille, BP 447, 1 rue A. Calmette, 59019 Lille Cedex, France; ^2^IFR142 Médecine Cellulaire et Moléculaire, Institut Pasteur de Lille, BP 447, 1 rue A. Calmette, 59019 Lille Cedex, France; ^3^Département de Neuroscience et de Physiologie Adaptatives, Bâtiment SN4, Université de Lille, 59655 Villeneuve d'Ascq, France; ^4^Bactéries Lactiques et Immunité des Muqueuses, Institut Pasteur de Lille/Institut de Biologie de Lille, BP 447, 1 rue A. Calmette, 59019 Lille Cedex, France

## Abstract

Endogenous intestinal microflora and environmental factors, such as diet, play a central role in immune homeostasis and reactivity. In addition, microflora and diet both influence body weight and insulin-resistance, notably through an action on adipose cells. Moreover, it is known since a long time that any disturbance in metabolism, like obesity, is associated with immune alteration, for example, inflammation. The purpose of this review is to provide an update on how nutrients-derived factors (mostly focusing on fatty acids and glucose) impact the innate and acquired immune systems, including the gut immune system and its associated bacterial flora. We will try to show the reader how the highly energy-demanding immune cells use glucose as a main source of fuel in a way similar to that of insulin-responsive adipose tissue and how Toll-like receptors (TLRs) of the innate immune system, which are found on immune cells, intestinal cells, and adipocytes, are presently viewed as essential actors in the complex balance ensuring bodily immune and metabolic health. Understanding more about these links will surely help to study and understand in a more fundamental way the common observation that eating healthy will keep you and your immune system healthy.

## 1. TOLL-LIKE RECEPTORS AT THE CROSS-ROAD BETWEEN IMMUNITY AND METABOLISM

The relationship between nutrition and the immune system has been a topic of study for much of the 20th century. Consequently, the dramatic increases in the understanding of the organization of the immune system and the factors that regulate immune function have supported the close concordance between host nutritional status and immunity.

### 1.1. The immune system: the concept of “immune recognition”

Classically, the mammalian immune system consists of innate and adaptive mechanisms that protect the host from environmental pathogens. Innate mechanisms function independently of previous exposure of the host to the infectious agent, and include mechanical barriers (e.g., skin, mucosal epithelium) and cellular components (e.g., mostly macrophages and neutrophils). In contrast to the innate immune system, the cellular (e.g., mostly B- and T-lymphocytes) and molecular basis of adaptive mechanisms relies on specific recognition of the invading agent and, like innate immunity, leads to the generation of immunological memory, that is, a property whereby an individual, after contacting an antigen for the first time, acquired the capacity to respond better and quicker upon reexposure to the same antigen.

Both innate and adaptive mechanisms are based on the general process of “immune recognition,” which has always been one of the main points of interest in immunology. For innate immunity, recognition is based on the use of germline-encoded receptors, whereas in adaptive immunity it involves somatically generated receptors. Nevertheless, beyond the different genetic nature of the receptors, the distinction between the two types of immune recognition—although useful in many ways—may obscure the heterogeneity of receptors and mechanisms of innate immune recognition.

The more recent advances in the field strongly suggest that the separation between innate and adaptive immunity may be too simplistic, notably at the cellular level. The actual concept is based on the existence of a continuum of immune cell populations highlighting the complex interplay between diverse cells of both innate and adaptive immune responses.

Below we will review the most recent findings in the field, focusing on the TLRs, which are now known to be the key regulators of both innate and adaptive immunities. Interestingly, we will indicate how the same TLRs have been reported to participate in metabolic integrity of a healthy individual.

### 1.2. Toll-like receptors: from innate to adaptive immunity

As mentioned above, the innate immune system allows a first-line protection to a broad variety of environmental pathogens independent of previous exposure to the infectious agent. It responds quickly and without memory capability, as opposed to adaptive immunity.

The innate immune system, through germline-encoded receptors, recognizes a limited set of conserved components of bacteria, parasites, fungi, or viruses, known as “pathogen-associated molecular patterns” (PAMPs). These receptors have therefore been called “pattern recognition receptors” (PRRs). Host cells express various PRRs that sense diverse PAMPs, ranging from lipids, lipopolysaccharides, lipoproteins, proteins, and nucleic acids. Recognition of these PAMPs by PRRs results in the activation of intracellular signaling pathways that culminate in the production of inflammatory cytokines, chemokines, or interferons, thus alerting the organism to the presence of infection [1].

Amongst others, PRRs include the members of the TLRs family [2], the nucleotide-binding oligomerization domain receptors (NOD-like receptors, NLRs) [3] and the retinoic acid-inducible gene-like helicases (RIG-like helicases, RLHs) [4]. Since their discovery, less than a decade ago, both TLRs and NLRs have been shown to be crucial in host protection against microbial infections but also in homeostasis of the colonizing microflora, as described in Section 1.3.

To date, the best characterized PRRs are the TLRs, a family of transmembrane receptors, the ligand-binding leucin-rich repeat domains of which interact with extracellular or membrane-enclosed (i.e., endosomal) intracellular PAMPs. Remarkably, TLRs are evolutionary conserved from plants to vertebrates. In mammals, 13 TLRs have been identified so far: 10 human (TLR1-10) and 12 murine (TLR1-9 and 11–13) receptors, of which some are homologous [5]. They are classified into several groups based on the type of PAMPs they recognize (considering the TLRs that we will mostly describe in this review: TLR2 senses bacterial lipoproteins, TLR4 senses lipopolysaccharide (LPS)). Two major signaling pathways are involved after TLR-ligand recognition. One pathway requires the adaptor molecule MyD88 while the other requires the adaptor Toll/IL-1 receptor (TIR)-domain-containing adaptor inducing IFN-*β* (TRIF), both involving translocation of NF*κ*B into the nucleus [6].

TLRs are broadly expressed in cells of the innate immune system such as macrophages, epithelial and endothelial cells, and in organ parenchyma cells, and have therefore specific roles in local innate immune defense [7].

Besides this first line of host defense towards microbial infections, the adaptive immune system is elicited later (around 4 to 7 days post-infection) and includes a specific and long-lasting immunity that is based on the rearrangement and the clonal expansion of a vast and random repertoire of antigen-specific receptors expressed on B- and T-lymphocytes (resp., B cell receptor: BCR and T cell receptor: TCR).

Interestingly, various TLRs are also expressed in cells of the adaptive immune system including B cells, mast cells, T cells, and dendritic cells (DCs), which are the key cells initiating the adaptive immune response. Indeed, TLR signals induce DC differentiation and cytokine production, consequently influencing the outcome of their interactions with T cells and therefore the subsequent development of the adaptive immune responses [8]. Recent in vitro studies demonstrate that TLR signals also trigger striking reorganization of the vacuolar compartments and affect MHC class II and membrane trafficking of DCs [9]. In addition, certain TLRs are also expressed in T lymphocytes, and their respective ligands can directly modulate T cell function. For example, TLR2, TLR3, TLR5, and TLR9 were shown to act as costimulatory receptors which enhance proliferation and/or cytokine production of TCR-stimulated T-lymphocytes [10]. Furthermore, specific subsets of T cells might selectively express different TLRs [11, 12]. Indeed, TLR4 is expressed by naïve CD4^+^ T cells in mice and in a CD25^+^ subset corresponding to regulatory T cells in humans. In addition, TLR2, TLR5, and TLR8 modulate the suppressive activity of naturally occurring CD25^+^CD4^+^ regulatory T cells. In B-lymphocytes, TLR signaling pathways also contribute to their activation and differentiation, mostly through the expression of TLR7 and TLR9 [13, 14].

Therefore, in addition to cells of the innate immune system, cells of the adaptive immune response, notably T- or B-lymphocytes and dendritic cells, express certain TLRs and respond directly to corresponding ligands in concert with TCR or BCR signals of lymphocytes. Thus in addition to their well-described role in innate immunity, TLRs are also crucial in shaping the adaptive immune response from its initiation to the development of immunological memory.

### 1.3. Toll-like receptors: role in mucosal immunity

Human and other mammalian mucosal surfaces are colonized by a vast, complex, and dynamic bacterial community. In human, the number of microbes associated with mucosal surfaces exceeds by 10 times the total number of body cells. This microbiota is constituted of more than 400 species, the collective genome of which being estimated to contain 100 times more genes than the human genome [15]. In the intestine, the microflora is in permanent contact and reciprocal interaction with the host cells and with nutrients, composing an extremely complex and highly regulated ecosystem.

The intestinal flora plays an important role in normal gut function and maintenance of the host’s health. It is established almost immediately after birth and is now considered to be essential in priming the immune system during ontogeny and in the development and maturation of both mucosal and systemic immune systems [16, 17]. Different factors contribute to the protective function of gut microflora such as (1) maintaining a physical barrier against colonization or invasion by pathogens, (2) facilitating nutrient digestion and assimilation, and (3) providing immunological surveillance signals at the gut mucosa-lumen interface. 

The microbiota is composed of potentially pathogenic bacteria besides numerous health-promoting nonpathogenic microorganisms. To control the resident colonizing microflora, as well as to fight pathogens, the human body has developed a variety of host defense mechanisms that in most cases effectively prevent the development of invasive microbial diseases [cf. Sections 1.1 and 1.2]. Commensals have been part of human microecology for millennia, however these “good bugs” are now less frequent or even absent in the microbial environment of our industrialized countries. Therefore, a link between the increasing incidence of allergies (Th2-driven pathologies) and the modern hygienic lifestyle has been suggested. This hypothesis, better known as “the hygiene hypothesis,” puts forward a dysregulation in the T helper (Th)1/Th2 balance but does not explain the increased incidence of several other immunological disorders such as inflammatory bowel diseases, multiple sclerosis, type 1 diabetes, and obesity, which are all primarily driven by Th1 cells [18, 19]. Recent findings have suggested that induction of regulatory T cells by certain microorganisms can prevent or alleviate such diseases [20]. Moreover, defects in such immunoregulatory processes, such as tolerance against the commensal microflora, have been shown to be associated to the pathogenesis of inflammatory bowel disease (IBD) [21].

Most interestingly, it was recently suggested that disruption of the mucosal barrier leads to the exposure of a multitude of commensal-derived TLR ligands that could interact with TLRs-expressing immune cells, consequently leading to potent inflammatory responses [22].

Paradoxically, nonpathogenic bacteria are thought to contribute to immune homeostasis, not only by maintaining microbial equilibrium but also by regulating the gut immune system. Indeed, commensal bacteria may directly influence the intestinal epithelium to limit immune activation. As mentioned before (cf. Section 1.1), commensal and harmful bacteria express conserved molecular features of microbes (i.e., PAMPs) necessary for stimulation of innate and/or adaptive immunity. Nevertheless, despite the fact that commensal bacteria *per se* are able to trigger PRRs, they do not induce inflammatory responses. To explain this apparent contradiction, it has been suggested that, whereas pathogenic bacteria can pass through the epithelial barrier and activate the TLR-dependent inflammatory cascade (notably by inducing NF*κ*B translocation), commensals would be sequestrated at the epithelial cell surface [23, 24].

Recent findings also reported a novel function of TLR signaling in intestinal homeostasis. Using knock-out mice, Rakoff-Nahoum et al. [25] demonstrated that the recognition of the commensal microflora by TLRs is required to dampen physiological inflammation present at the steady state, explaining why any disequilibrium in this signaling pathway will lead to inflammatory bowel diseases. Recent studies in mice also showed that in vivo ablation of NF*κ*B activation in colonic epithelium caused severe chronic intestinal inflammation [26], demonstrating that NF*κ*B signaling is a critical regulator of epithelial integrity and intestinal immune homeostasis. Moreover, several reports indicated that commensals are able to dampen intestinal inflammation by inhibiting the NF*κ*B signaling pathway. Neish et al. [27] first showed that avirulent *Salmonella* abrogates the inflammatory cascade by inhibiting ubiquitination and degradation of I*κ*B, thus blocking the transactivation of NF*κ*B-mediated genes. More recently, Kelly et al. [28] identified an interesting mechanism by which commensal flora may regulate host inflammatory responses and maintain immune homeostasis, more particularly by promoting nuclear export of NF*κ*B subunit relA, through a PPAR-*γ*-dependent pathway. Another possible mechanism to inhibit inflammatory responses at mucosal sites is the generation of tolerance to a subsequent stimulation from bacterial products. Otte et al. [29] reported that repeated contact with bacterial components (e.g., lipopolysaccharides) down-regulated epithelial TLR expression, and inhibited intracellular signaling through TLRs by up-regulating Tollip, an inhibitor of TLR-mediated cell activation. These data collectively suggest mechanisms whereby inflammatory responses induced by commensal bacteria are inhibited to create and maintain a state of “immunological silence” at the intestinal mucosa.

Besides TLRs, other PRRs have recently been shown to be involved in these processes, notably members of the NOD-like receptor (NLR) family. NLRs can detect bacterial components such as muramyldipeptide (MDP) (recognized by NOD-2) and muropeptides containing mesodiaminopimelic acid (recognized by NOD-1).

It is known that NOD signaling involves the activation of NF*κ*B pathway, but surprisingly, mutations in the *CARD15* gene, affecting NOD-2 function, increase the susceptibility to Crohn’s disease [30]. Recent papers reported that NOD-2-deficient antigen presenting cells (APCs) showed increased NF*κ*B activation and IL-12 production upon exposure to peptidoglycan (PGN), a TLR2 ligand that could also give rise to MDP, and the specific ligand of NOD-2. In addition, it was shown that MDP could down-regulate the IL-12 response of normal APCs to PGN [31]. Using transgenic mice, the same group showed that mice overexpressing NOD-2 exhibited greatly decreased IL-12 responses to systemic administration of PGN but not to LPS and are partially resistant to colitis induction. These results brought new evidence that defects in the NOD-2 signaling will contribute to inflammatory bowel diseases by leading to excessive TLR2-dependent inflammatory responses [32]. The authors hypothesized that mucosal APCs are normally exposed to PGN derived from commensal bacteria leading in normal individuals to innate immune responses. This response would be reasonably weak, owing to NOD-2 modulation and also through the induction of regulatory responses. In case of NOD-2 signaling defects, the TLR2-dependent inflammatory response could not be controlled, therefore leading to mucosal injury.

It is now well accepted that homeostasis versus chronic intestinal inflammation is determined by the presence or absence of appropriate control mechanisms that could be linked to a balance between protective (“good”) and aggressive (“bad”) luminal bacteria. Indeed, recent findings reported notable influence of the microbiota composition on the incidence of emerging pathologies such as inflammatory bowel diseases (IBD) and obesity. Metagenomic analysis indicates that the microflora of IBD patients is unstable and presents a reduced complexity of the bacterial phylum Firmicutes. Conversely, a shift in the ratio of Bacteroidetes to Firmicutes has been observed in obese patients as well as in leptin-deficient obese mice (*ob/ob*) [33, 34]. Thus, the outcome of severe and critical illnesses seems to be strongly related to environmental factors and their interaction with the innate immune system. As we described above, cooperative as well as competitive interactions may occur between different microbial ligands via TLRs and NODs or via other components of the innate immune system, leading to either protective or deleterious responses.

### 1.4. Toll-like receptors: role in metabolism

Interestingly, in addition to playing a crucial role in immunity, some of the mammalian TLRs have been described to regulate bodily energy metabolism, mostly through acting on adipose tissue. This has recently opened new avenues of research on the role of TLRs in pathologies related to metabolism, such as obesity, insulin resistance, or atherosclerosis.

#### 1.4.1. TLRs sense lipids and lipids act on TLRs

As previously discussed (Section 1.2), TLRs can recognize several types of components, among which lipids. It has been shown that some agonists of TLRs contain a lipid moiety comprising saturated fatty acids in acetylated form and which is essential for the agonistic activity. This is the case for the lipid A moiety that supports most of the biological activity of LPS, the ligand of TLR4 [35], or for the lipopeptides which activate TLR2. Interestingly, if the acetylated saturated fatty acids of these TLR agonists are deacetylated or replaced by unsaturated fatty acids, the agonists lose their activity or act as antagonists [36, 37].

Starting from these observations, Lee and collaborators postulated in 2001 that fatty acids could possibly directly modulate TLR activation and expression of target gene products [38]. These authors reported that saturated fatty acids were able to induce the activation of TLR2 and TLR4, whereas unsaturated fatty acids inhibited TLR-mediated signaling pathways and gene expression (reviewed in [39]). Both activation of MyD88-dependent and TRIF-dependent TLR signaling pathways were achieved by saturated fatty acids. Inversely, unsaturated fatty acids suppressed NF*κ*B activation induced by LPS, the TLR4 agonist. Importantly, this inhibitory effect of PUFAs on LPS-induced inflammation was verified with blood peripheral monocytes harvested from people given a diet containing fish oil, a major source of *n*-3 PUFAs. Finally, this dichotomy of effects depending on fatty acid type was not only observed with macrophages but also with dendritic cells, implying that lipids can affect cells of both the innate and the adaptive immune systems. Through TLR4 activation, saturated fatty acids could upregulate the in vitro expression of costimulatory molecules (e.g., CD40, CD80, and CD86), MHC class II and cytokines (e.g., interleukin (IL)-6 and -12) on DCs, increasing therefore their capacity to activate T cells. Again, this activation was inhibited by adding unsaturated fatty acids, or when using a dominant negative mutant of TLR4. However, opposite effects were obtained in vivo and dyslipidemia resulting from high-fat feeding was hypothesized to impair TLR-induced activation of mouse dendritic cells [40]. Indeed, a defect in CD8*α* ^−^ myeloid dendritic cells was observed in mice after high-fat diet, leading to impaired Th1 and enhanced Th2 responses, and to increased susceptibility to pathogens. Recently, Shi et al. postulated and demonstrated that TLR activation achieved by fatty acids from diet origin led to proinflammatory cytokine production, and thereby promoted insulin-resistance [41]. Nutritional saturated fatty acids potently stimulated IL-6 or tumour necrosis factor (TNF)-*α* mRNA expression in macrophage-like cells, whereas food-derived polyunsaturated fatty acids had no effect alone but inhibited saturated fatty acid-induced TNF-*α* mRNA expression. Additionally, macrophages isolated from TLR4-deficient mice showed blunted cytokine expression in response to saturated fatty acid treatment.

Compared to TLR4, the direct interaction of TLR2 with lipids is less documented, but the existence of a link between lipids and TLR2-signaling has been suggested. Activation of TLR2 is mainly involved in promoting vascular inflammation and the development of the atherosclerotic plaque. Inactivation of TLR2 expression by knockout technology was shown to protect atherosclerosis-susceptible mice from the development of disease [42]. Indeed, TLR2 forms complexes in lipid rafts with CD36, a membrane receptor which binds fatty acids and facilitates their transfer into the cells and which is involved in atherosclerosis progression [43]. Amazingly, CD36 was described as facilitating TLR2 signaling [44]. Thus the interaction of lipids with different partners of the TLRs family could take diverse aspects other than a direct interaction.

In conclusion, beside its primary function in alerting the immune system to the presence of pathogenic microorganisms, TLRs could also sense pathological levels of lipids. In this context, it is interesting to notice that LPS presented an anorexigenic effect that was blunted in TLR4-deficient mice [45], and that TLR4-deficiency could eventually lead to change in eating behaviour, either increasing or decreasing food intake [41, 46, 47] (cf. Section 1.4.3). Therefore, it is tempting to speculate that detection of abnormal levels of dietary lipids by TLRs could participate to the sensing of the energy state of the body and to the subsequent control of food intake. However, more studies are still needed to clarify the controversial results concerning food intake status in TLR4-deficient mice before concluding on a potent role of TLRs in the regulation of food intake. Regarding this, one should particularly consider the involvement of LPS in these different models.

TLRs are widely distributed in the body notably in the brain where these receptors are expressed by glial cells [48, 49] and by endothelial cells forming the vessels that irrigate the brain [50]. It has recently been reported that TLR2 and TLR4 are expressed by cortical neurons [51] and interestingly, these neuronal TLRs appeared to be insensitive to bacterial motifs despite being reactive to endogenous products such as the heat shock protein 70. To our knowledge, the precise analysis of TLR activation in the hypothalamus (the brain region mostly dedicated to food intake and body weight control) has not yet been achieved. This would be of fundamental importance to possibly envisage the participation of TLRs in the central control of energy homeostasis.

#### 1.4.2. TLRs are expressed on adipose cells

Insights obtained over the last years have shown adipose tissue to be a true immunocompetent organ and adipocytes as intricate components of the innate immune system. Indeed, adipocytes produce numerous inflammatory molecules such as IL-6 or TNF-*α* [52, 53]. In addition, leptin, the champion of adipocyte-specific factor, has been shown to play an essential role in both innate and adaptive immune responses [54]. Besides, adipocytes and macrophages (the prototypes of cells involved in innate immunity) were recently described to originate from a common ancestral progenitor and to share several features [55–57]. Macrophages express some adipocyte-specific gene products such as ap2, while adipocytes secrete macrophage-specific gene products such as IL-6 or TNF-*α*. This common gene expression results in some analogous functional activities, such as lipid accumulation by macrophages in atherosclerotic lesions or phagocytic capacities exhibited by adipocytes towards certain pathogens, thus revealing an apparent coordinated activity between these two cell-types during the course of an innate immune response.

An additional similarity between adipocytes and macrophages was further revealed with the reporting of the expression of TLR4 (the TLR mostly known to sense LPS) by the murine preadipose cell line 3T3-L1 [58]. Interestingly, LPS-treated adipose cells secrete increased amounts of TNF-*α*, and subsequently express higher levels of TLR2. Recently, the presence of functional TLR2 and TLR4 was reported on human adipocytes isolated from subcutaneous fat tissue [59], and several TLRs (TLR1 to 9) were found on adipocytes derived from murine adipose tissue [60, 61]. Activation of adipocytes via TLRs (mostly TLR4) results in synthesis of proinflammatory factors such as TNF-*α* or IL-6, and of chemokines such as CCL2, CCL5, or CCL11 [58, 59, 62]. Conversely, adipocyte-specific knockdown of TLR4 (e.g., shRNAi for TLR4 in 3T3-L1 cells; or adipocytes from TLR4-deficient mouse) prevented cytokine expression induced either by LPS or by saturated fatty acids. Finally, adipocytes isolated from diet-induced obese mice or genetically obese animals (*ob/ob* or *db/db*) exhibited increased TLR expression [41, 61, 63], together with higher cytokine production upon stimulation [61].

#### 1.4.3. TLRs, fatty acids and the metabolic syndrome

The observations summed up above may indicate that the triad “adipocyte-macrophage-TLR4” might be involved in the inflammatory process occurring in obesity. Indeed in the obese state, a marked infiltration of macrophages is observed within the adipose tissue. Suganami et al. showed that lipolysis and proinflammatory cytokine production were reduced when adipocytes isolated from obese mice were cocultured with TLR4-deficient macrophages, compared to wild-type macrophages [64]. Thus the *duo* “saturated fatty acids plus TLR4” might be responsible for the amplification of inflammation occurring in obesity. In this vicious circle, increased amount of saturated fatty acids (provided either by high-fat feeding or adipocyte lipolysis) could serve as naturally occurring ligands for TLRs (mainly TLR4), resulting in the activation of both adipocytes and macrophages to produce proinflammatory products, ultimately leading to the development of the metabolic syndrome.

This seems to be the case, since mice genetically deficient in TLR4 or in CD14 (a coreceptor for TLR4) were reported to be of “ideal body type” when fed on regular chow, having increased bone mineral content, density, and size, as well as decreased body fat [65]. Moreover, these mice do not become obese with age, unlike many strains of laboratory wild-type mice. This “perfect” phenotype of low adiposity and strong bones, with normal activity and fertility was baptized as “The Adonis phenotype” and the concept is currently further explored for its potential in the treatment of obesity.

However, this approach has to be considered with caution since contradictory results have been obtained with high-fat-fed TLR4-deficient mice. Indeed, while some reports described no effect on body weight [46, 64, 66], other authors described an increased body weight [41] or, in contrast, a protection against diet-induced obesity [47]. Similarly, adiposity and food intake were either reported to be unchanged, increased, or decreased in TLR4-deficient animals [41, 46, 47, 64]. Even though these studies were conducted on mice with different genetic backgrounds or obtained with different TLR4-mutating strategies and using different feeding protocols (e.g., diet composition and timing), and despite the discrepancies obtained on body weight and adiposity levels, they all revealed a marked improvement in insulin sensitivity in the TLR4-deficient mouse as compared to the WT animals. Therefore TLR4, being expressed in most tissues of the body—including the insulin-sensitive ones such as adipose tissue (cf. Section 1.4.2), muscle, and liver [47]—and due to its activation by lipopolysaccharide and saturated fatty acids, which are both inducers of insulin-resistance, appears to be an essential mediator of bodily insulin-resistance. Interestingly, it has been suggested that both TLR2 and TLR4 might be involved in hepatic lipid trafficking and storage [67], yet their precise role in fat accumulation in the liver still needs to be determined.

Along the same lines another PRR, known as receptor of advanced glycation end products (RAGE), has recently been put in the spotlight. The interaction between RAGE and its ligands, advanced glycation end products (AGEs) such as lipids and nucleic acids resulting from oxidative stress and hyperglycemia [68], activates NF*κ*B, which leads to transcription of proinflammatory factors [69]. Even if their relevance for obesity is still unclear, AGEs were shown to accumulate in pathological conditions such as diabetes or under particular life-style habits such as unhealthy diet consumption [70]. Furthermore, RAGE and its ligands have been implicated in multiple chronic inflammatory diseases such as atherosclerosis and diabetes [71]. Interestingly, alike the canonical Toll receptors, RAGE is expressed in macrophages [72], and several experimental evidences strongly support a role for RAGE in innate immune responses [73].

## 2. ENERGETIC DEMANDS OF THE IMMUNE SYSTEM: A SPECIAL TRIBUTE TO DIETARY LIPIDS AND TO GLUCOSE

Despite the apparent independence between the fields of immunology and nutrition, myriad observations, some quite old and some quite new, clearly show that the immune system cannot function under circumstances of malnutrition, whether over- or undernutrition [74].

Indeed, lipids consumed in the diet (e.g., fatty acids, cholesterol, or fat-soluble vitamins), glucose, or oligoelements (e.g., zinc, copper, and iron) deeply affect the immune system. Revealing this strong dependence of the immune system upon nutrition, is the fact that nutritional deficiencies are presently considered to be the most common cause of secondary immunodeficiencies in humans.

### 2.1. Historical backgrounds: importance of zinc and lipids

Historically, the model of zinc-deficiency states as the best characterized nutritional-immunological paradigm. Zinc-deficiency was shown to impact on B-cell lymphopoiesis and to induce potent atrophy of the thymus, subsequently leading to a decline in the number of peripheral T-lymphocytes, both in a murine model of zinc deficiency and in zinc-deficient humans [75–77]. In fact, this anatomical link between nutrition and immunology reflected by the description of the thymus as the “barometer of nutrition” was recognized long before the thymus was found to be of key immunological importance. The crucial role of zinc or other oligoelements, in the immune system, has been extensively described in excellent reviews which we invited the readers to go through [78, 79].

Considering the influence of dietary lipids on immune function, it is rather surprising that this relation was only seriously investigated during the past two decades. It is clear from whole-animal studies that obesity and consumption of high fat-diets, particularly saturated fat, depress both innate and adaptive immunocompetences by affecting the activity of immune cells such as macrophages, dendritic cells, or T lymphocytes, thereby enhancing the risk for serious infection and cancers.

The relationship between lipids and immune response is complex, multifactorial, and still poorly understood. Beside individual susceptibility, linked to genetic factors, the deleterious effect of fat depends largely on the quantity and the quality of the lipid species consumed. Classically, saturated fatty acids are presented as “bad lipids” by increasing total cholesterol and as being associated with inflammation and increased cardiovascular events. In contrast, unsaturated fats and particularly omega-3 fatty acids are considered to be “good lipids” by decreasing cholesterol and by preventing adverse symptoms of metabolic syndrome such as insulin resistance and inflammation. Exhaustive reviews treating the effects of fat ingestion on molecular and cellular aspects of immunity have been published [80–82], and will not be further developed here. Therefore, we restrict ourselves to present some selected aspects of these interactions, before discussing some examples of the consequences of high-fat feeding on immune reactivity in the light of some of the results we recently obtained.

Besides modulation of immune responses via interactions with Toll-like receptors at the surface of immune cells (see Section 1.4), lipids appear essential in the performance of immune cells both as energy suppliers and as constituents of the membrane architecture. The lipids involved originate either from the white adipose tissue or directly from nutrition.

In case of a foreign attack, energy needs to be delivered very rapidly, allowing an immediate reaction of the body. An essential contribution of the adipose tissue is then to supply immune cells with fatty acids, which will serve as fuel, as well as lipid-based messenger molecules. Indeed, arachidonic acid and docohexanoic acid, two lipid-derived messenger molecules originating from polyunsaturated fatty acids (PUFAs), are key factors in innate immune processes, since they are the precursors for prostaglandins and leukotrienes, both largely involved in inflammation [83]. This may also explain why lymph nodes are always embedded within fat depots, thus emancipating the immune system from competition with any other tissue [84]. In vivo, following a local immune activation, spontaneous lipolysis is observed specifically in the adipocytes surrounding lymph nodes, implying the active participation of these adipose cells in local and transient immune responses [85]. This close interaction between adipose and lymphoid tissues was verified in some chronic pathologies where selective expansion of perinodal adipose depots is evidenced, while other depots are depleted [86]. This is the case of Crohn’s disease, which affects the alimentary tract and in which only fat depots associated to mesenteric lymphoid tissue expand [87, 88]. It was also observed in long-term treated HIV patients, where change of adipose tissue distribution (HARS; HIV-associated adipose redistribution syndrome, [89]) could be due to the prolonged activation of perinodal adipose tissue, resulting in enlargement of node-containing depots, at the expense of nodeless depots [86, 90].

Moreover, lipids are major components of cell membranes, and combinational associations of different lipid species will generate microheterogeneity in cell membranes, leading to the formation of microdomains, termed rafts [91, 92]. Differences in lipid raft composition and organization have been associated with differences in T cell signaling and in synapse formation between APC and T cells [93–95]. Furthermore, a differential implementation of rafts has been demonstrated between T helper (Th)1 and Th2 cells, indicating that the regulation of T cell signaling and activation by lipid diet may be crucial in Th1/Th2 cell orientation [96]. The lipid-content of the membrane of dendritic cells and lymphoid cells in nodes containing depots was shown to correlate well with that of the adjacent adipocytes [97]. Conjointly, besides *de novo* synthesis from carbohydrates, fatty acids deposited in adipose tissue can originate from dietary sources. Thus, any diet-induced variation in lipid composition of fat depots may influence directly the membrane organization of immune cells and result in impaired functionality. Indeed, it was shown that diet has a marked impact on the lipid composition of cell membranes, leading to changes in fluidity and organization [98]. In particular, dietary (*n*-3) PUFAs alter T cell membrane microdomain composition and may therefore influence signaling complexes and modulate T cell activation in vivo [81].

### 2.2. Role of glucose in the immune system: why, when, and how?

As we will describe in the last section (cf. Section 3.1), fluctuations in blood glucose occur in inflammatory diseases such as obesity, diabetes, and insulin resistance, in which gut microbiota might play an active role. We will show now that, in addition to lipids (cf. Section 2.1), glucose should be considered the quantitatively most important fuel to fulfil the energy requirement of immune cells, therefore it is likely involved in the immune alterations associated with obesity or diabetes.

#### 2.2.1. Role of glucose in the immune system: why?

The immune system—both innate and adaptive—is essential to prevent or limit infection but is equally important in the overall process of repair and recovery from any type of injury. As described in Section 1.3, the immune system also participates in the control of the resident colonizing microflora which is essential to the establishment of an “immunologic and metabolic health.” To exert this variety of fundamental regulatory processes—some of which being highly energy demanding—immune cells from the innate and the adaptive immune systems utilize numerous extracellular molecules and signals as fuels [99–102]. The exact nature of the energetic demands and how these are met will differ among immune cells and the nature of the required response; for example, whether proliferative/secretory (B- or T-lymphocytes) or nonproliferative/secretory (macrophages or neutrophils) will be important. However, any type of response will place large bioenergetic demands on all immune cells. In addition to glutamine, ketone bodies, or fatty acids, glucose should be considered the most quantitatively important fuel for immune cells.

Indeed, early studies using lymphocytes stimulated with B- or T-specific mitogens (such as pokeweed mitogen (for B cells), concanavalin-A, or phytohemagglutinin-A (for T cells)) revealed the importance of glucose uptake and catabolism in providing energy for their proliferative, biosynthetic, and secretory activities [103–107]. Within 1 hour of stimulation, mitogen-induced lymphocyte activation led to an increase in glucose consumption, mostly metabolized to lactate, highlighting a rapid enhancement of glycolysis following lymphocyte activation. Additionally, other pathways of glucose utilization were also shown to be induced during lymphocyte stimulation, such as the pentose phosphate pathway which peaked at 48 hours after stimulation, coinciding with the maximal protein and RNA synthesis accompanying lymphocyte blastogenesis [108].

Later, the crucial role of glucose in lymphocyte activation was also reported to be expandable to cells of the innate immune system like macrophages [109] and neutrophils [110]. Although the capacity for rapid cell division does not apply to these cell types, which are terminally differentiated and have little capacity for cell division, macrophages and neutrophils have a large phagocytic capacity (requiring a high rate of lipid turnover and synthesis) and a large secretory activity in which glucose was shown to be most likely involved.

To conclude, generating an efficient and effective immune response involves large increases in cellular proliferative, biosynthetic, and secretory activities, processes which all require high energy consumption. As mentioned, adaptive as well as innate immune cells must be able to rapidly respond to the presence of pathogens, shifting from a quiescent phenotype to a highly active state within hours after stimulation. For that purpose, cells must dramatically alter their metabolism in order to support these increased synthetic activities based on extracellular signals as fuels, amongst which glucose is the most essential one.

#### 2.2.2. Role of glucose in the immune system: when?

Lymphocyte development is tightly controlled, starting from multipotent medullary progenitors to mature lymphoid cells in the periphery. For T cell lineages, that were more extensively studied for their glucose metabolism than the B-cell lineages, a crucial checkpoint in T-lymphocyte development occurs in the thymus where the Notch and the IL-7 receptor (IL-7R) signaling pathways both maintain cell viability and promote thymocyte differentiation [111, 112]. Interestingly, it has been shown that both pathways are important for glucose metabolism in T cells, notably via Akt/PKB activation [113, 114]. Resting T cells will later exit the thymus and enter peripheral circulation as small quiescent cells. These resting cells consume glucose and other nutrients at a low rate, sufficient to maintain normal housekeeping functions. Even to insure this basal metabolic rate, T cells require extracellular signals from, for example, cytokines as well as low-level stimulation through the TCR. In the absence of such signals, T cells will reduce their capacity to import glucose to levels below those necessary to maintain cellular homeostasis [107, 115, 116]. Thus, the metabolism of resting lymphocytes is limited by the availability of trophic signals rather than the availability of nutrients, such as glucose [117]. Once T cells are activated by mitogens or antigens, the energy-demanding processes are activated as described in Section 2.2.1. In order to approximately double their resting size and enter a program of rapid proliferation while differentiating from a quiescent to a highly secretory state, activated T cells will strikingly increase their glucose consumption, a demand mostly met through glycolysis [107].

Interestingly, it was recently reported that increased extracellular concentrations of glucose can protect neutrophils from apoptotic death and that this protective effect is correlated with the rate of glucose utilization by the cells [118]. Apoptosis is an important feature of neutrophil biology and prevention of neutrophil death by high glucose concentrations might be seen as beneficial since these cells are key components of the innate immune response.

#### 2.2.3. Role of glucose in the immune system: how?

Recently, a combination of independent and complementary studies has provided molecular insights into the regulation of energy metabolism in immune cells, involving the coordination by signal transduction pathways which act directly onto the modulation of nutrient uptake and metabolism.

First of all, both the major glucose-transporter (GLUT) proteins and the insulin receptor (InsR) were shown to be expressed on immune cells (e.g., monocytes/macrophages, neutrophils, and B- and T-lymphocytes) [119–121]. Those receptors are functional since they are responsive to both immune stimulation and insulin [122].

The pattern of GLUT upregulation differs among different types of immune cells. For example, differentiation of monocytes to macrophages is associated with an increased expression of GLUT3 and GLUT5, even if their precise physiological role in macrophages still remains uncertain [123]. Regarding insulin-stimulating glucose transport, it was shown that physiological doses of insulin led to increased expression of GLUT3 and GLUT4 in monocytes and B-lymphocytes [124]. In contrast, insulin did not alter GLUT expression neither in resting T cells nor in neutrophils [122–124], despite activating the insulin-signaling pathway [125]. Nevertheless, in vitro mitogen- or LPS- (the ligand for TLR4) stimulation of immune cells enhanced the expression of membrane GLUT isoforms, mainly GLUT1, 3, and 4 [122–124]. Interestingly is to note that the increase in GLUT1 levels upon stimulation was observed with all cell types (e.g., monocytes/macrophages and T- and B-lymphocytes), likely suggesting that GLUT1 might be the isoform which ensures the provision of glucose for the basic metabolic needs [126]. Important also is the observation that GLUT3 and GLUT4 and GLUT isoforms with higher affinity for glucose were strongly overexpressed on activated T- and B-cells, therefore allowing immune cells to compete for glucose when concentrations in the surrounding environment are very low. This is particularly important for lymphocytes, which have low energy-storage capacity [99] and, as we discussed before, are high energy demanders especially in conditions of activation.

In addition to the increased expression of GLUT isoforms upon immune stimulation (i.e., by mitogen or LPS), insulin withdrawal on immune cells was also reported to modulate GLUT expression, notably GLUT3 and GLUT4. It has been proposed that expression of the Insulin receptor is essential for immune cell division, size, and survival [127] and that IL-7 would be essential in this process [128].

Secondly, regarding the signaling pathways that modulate the glucose uptake and metabolism of immune cells, it was reported before that treatment of B- or T-cells with inhibitors of phosphatidylinositol3-kinase (PI3-K) blunted the ongoing increase in cell size, and therefore the subsequent proliferation, probably as a result of a block at a critical early growth checkpoint [129]. This observation further supports a key role for glucose metabolism in immune cells.

In T cells, it is known that ligation of the costimulatory receptor CD28 activates the PI3-K/Akt pathway [130], similarly to the binding of insulin to its receptor [131]. Therefore, CD28 was suggested to be a good candidate for regulating T cell metabolism [116]. Indeed, upon CD28 stimulation, T cells increase GLUT expression, glucose uptake, and glycolysis and these effects are dependent on PI3-K activity [116]. Additionally, CTLA-4, an inhibitory receptor with opposite effects on T cell activation, can inhibit CD28-induced increases in glucose metabolism [116].

The precise signaling mechanisms by which growth factors or cytokines (glucose, insulin, and IL-7 as the most important ones) prevent atrophy and promote cellular metabolism in immune cells still remain uncertain. Nevertheless, PI3-K and mammalian target of rapamycin (mTOR) have been shown to simulate cellular metabolism and are activated by a variety of growth stimuli such as glucose, insulin, and IL-7. PI3-K and its downstream signaling molecule Akt can promote glucose uptake and metabolism [116] while mTOR is critical in promoting protein-efficient translation and inhibiting protein degradation [132].

Regarding IL-7, an immune cytokine essential for survival, cell size, and T cell activation, it was shown to maintain glucose metabolism in vitro. Indeed, the addition of IL-7 to T cell cultures was found to be sufficient to maintain glucose metabolism to approximately normal levels. In addition, like for insulin/glucose, the trophic effect of IL-7 requires PI3-K and mTOR activities [128].

In conclusion, when considering the signaling pathways involved in glucose metabolism in immune cells, it is generally accepted that glucose uptake and metabolism are promoted by PI3-K and its downstream signaling molecule Akt (both in T- and B-lymphocytes). mTOR appears to be more critical in favoring efficient protein translation and inhibiting protein degradation. Interestingly, the crucial role of IL-7 on T-lymphocyte homeostasis (in mice and human)—known for a long time—was demonstrated to depend upon these metabolic pathways since IL-7, alike insulin, promotes T cell survival and size in a PI3-K/Akt and mTOR-dependent manner.

## 3. SELECTED EXAMPLES OF THE IMMUNE REACTIVITY OF METABOLICALLY ALTERED ORGANISMS

After having described the intricate relations between the immune system, selected nutrients such as glucose or lipids, and the endogenous microflora, we will illustrate below how malnutrition (mostly overnutrition) can affect immunocompetence.

### 3.1. Obesity, diabetes, and immune dysfunction

The incidence of obesity and associated comorbidities—such as type 2 diabetes, insulin resistance, and cardiovascular diseases—is reaching worldwide epidemic proportions [133–135]. This pathology is the result of an imbalance between caloric intake and energy expenditure, resulting in excess energy storage, mostly due to genetic and environmental factors. Among the environmental factors thought to play an important role in obesity, we should count the increased consumption of energy-dense and micronutrient-poor foods, that is, processed food is usually high in starches, added sugars, and added fats [136, 137].

After a meal, fatty acids and glucose enter the blood. As shown above, both factors greatly influence immune homeostasis and reactivity. In obesity, the body is literally soaked in excess fat and glucose, likely participating to the profound alterations of immune responsiveness—innate and adaptive—occurring in the obese state.

Indeed, macrophages accumulated proportionally to adipocyte size and numbers within the white adipose tissue of obese mice. In addition, macrophages from this “obese adipose tissue” displayed impaired functionality with a reduced phagocytic capacity and a defective oxidative burst [138, 139]. More generally, several independent epidemiological studies reported that obese individuals have increased susceptibility to systemic infections. The obese patients are more prone to develop infectious complications after surgery [140], and a positive correlation between body mass index (i.e., weight in kilograms divided by height in square meters) and nosocomial diseases has been reported [141]. Moreover, up to 50% of obese persons develop cutaneous infections and display reduced wound healing capabilities [142–145]. In longitudinal studies, the incidence of lower respiratory tract infections was significantly higher in obese infants than in nonobese infants [145]. Chandra [146] reported that obese children, adolescent, and adults exhibited variable impairment of cell-mediated immune responses in vivo and in vitro as well as a reduction of intracellular bacterial killing by polymorphonuclear (PMN) leukocytes.

This marked impairment of the immune system associated with human obesity has also been reported in several animal models. Obese dogs have a decreased capacity to resist *salmonella* infection and canine distemper virus [147]. In addition, these obese dogs have shortened average survival time after distemper infection and the incidence of paralytic encephalitis was significantly increased [148]. In rodents, it was shown that the obese zucker rats have an increased susceptibility to *Candida albicans* infections [149], whereas obese leptin-deficient *ob/ob* and leptin-resistant diabetic *db/db* mice display an impaired response to *Listeria monocytogenes* [150]. As in human obesity, obese animals present a delayed wound healing associated with increased polymorphonuclear cell infiltration [151]. In addition, both T- and B-cell-mediated immune responses were reported to be impaired in obese *ob/ob* and diabetic *db/db* mice [152, 153].

Finally, obesity is also characterized by an imbalance of the cytokine network, resulting in a low-grade systemic inflammatory status described in both obese humans and animals [154]. The inflammatory cytokines IL-6, IL-1, and TNF-*α*, abnormally elevated in obesity, mostly originate from the activated macrophages infiltrating the white adipose tissue [138, 139, 155].

Thus, obesity is presently viewed as an inflammatory disease, referred to as “obesitis,” affecting both innate and acquired immune systems [156]. We described in Section 1.4.3 how TLR4 might be involved in the inflammation occurring in the obese state, and recent studies reported a protection of high-fat fed mice against insulin resistance and vascular inflammation in TLR4-deficient mice compared to WT animals [41, 46, 47, 64]. In human, some associations between TLR4 polymorphism and vascular inflammation, artherosclerosis and clinical diabetes, have also been published [157].

Although many research groups have studied the immune system of obese individuals or animals, there is still scarce information regarding the effects of obesity on dendritic cells (DCs), despite their essential role in innate immunity and in the induction and regulation of antigen-specific adaptive responses [158].

Therefore, we recently characterized DCs in the model of obese leptin-deficient *ob/ob* mice [159]. Leptin is an adipocyte-derived cytokine, secreted proportionally to the amount of fat, originally characterized for its capacity to finely regulate body weight [160]. Indeed, the complete congenital absence of leptin leads to a syndrome of intense hyperphagia and morbid obesity both in humans and rodents, which can be reverted by administration of the recombinant molecule [161]. Interestingly, subsequent studies further demonstrated that leptin intervenes in both innate and adaptive immunities. Leptin promotes activation of monocytes/macrophages chemotaxis and activation of PMN cells, development and activation of natural killer (NK) cells, and regulation of T cell responsiveness [162]. Therefore, due to these multiple functions of leptin, mice lacking the functional protein (e.g., the *ob/ob* mice) present a broad range of endocrine and immune alterations.

Among these immune alterations, we demonstrated that despite displaying normal phenotypic and functional characteristics, both homeostasis and functionality of DCs were disturbed in *ob/ob* mice. Indeed, DCs from *ob/ob* mice were less potent in stimulation of allogenic T cells in vitro, likely due to the increased secretion of immunosuppressive cytokines. Moreover, we showed altered in vivo homeostasis of epidermal DCs in *ob/ob* mice, which was not due to a migratory defect and which could be restored by intradermal administration of leptin [159].

Along those lines, we also reported the impairment of immune cells as a consequence of high fat diet- (HFD-) induced weight gain (a more physiological model of obesity than the *ob/ob* model) in a study using mice transgenic for a TCR recognizing a peptide derived from ovalbumin [163]. The study showed that T cell reactivity was impaired by excess of fat feeding, but amazingly the expression of this effect was dependent on whether T cells are naïve or antigen-experienced. Indeed, T cells from HFD-fed naïve transgenic mice exhibit a strong proinflammatory profile when stimulated in vitro with mitogen or antigen, implying that these cells likely participate in the low-grade systemic inflammation observed in overweight and obese patients. Inversely, antigen-experienced T cells (from ovalbumin-immunized HFD-fed mice) presented a marked defect in proliferative capacity, together with a shift towards a typical Th2 cytokine secretion profile. Dendritic cells apparently played a pivotal role in the Th polarization and impaired maturation was associated with a Th2 immune deviation [164]. We did observe that DCs were defective in their capacity to present antigens to T cells in HFD animals. This Th2-biased immune response could be involved in the high incidence of infection reported in obese patients, and in hyporesponsiveness to some vaccination trials [140–146].

Altogether, we demonstrated for the first time that the immune deficiency observed in leptin-deficient obese mice, and maybe in other types of obesity, was associated with an impairment of dendritic-cell function, the key immune cell that bridges innate and adaptive immunities.

As stated in the introduction to this last section, fat and glucose controls are linked: obese people develop insulin resistance and then diabetes, conditions in which glucose uptake and production are impaired due to defective insulin action [133–135]. In Section 2 of our review, we showed how glucose transport and metabolism in immune cells are sensitive to insulin. In addition, we showed that glucose is a major fuel used by immune cells, therefore any variation in blood glucose concentration will likely affect immune responsiveness. Indeed, it was reported that acute, short-term hyperglycemia affects all major components of innate and acquired immunities, consequently leading to reduced defense against infection [165] and initiating a cascade of pathological events resulting in the activation of NF*κ*B [166].

In diabetes, where insulin action is defective and hyperglycemia chronic, immune T cell functionality is impaired with reduced ability to produce IL-2 [167] and to proliferate in response to mitogenic or antigenic signals [168]. Furthermore, neutrophils from diabetic patients showed impaired respiratory-burst activity [169]. Additionally, a pioneering study by Van den Berghe et al. [170] reported that patients receiving intensive insulin therapy had a significantly reduced rate of infections and were less likely to have elevated markers of inflammation.

Nevertheless, despite the fact that increased susceptibility to infections affects the morbidity and mortality of diabetic patients—which is of critical clinical importance—little is known about how diabetes precisely impair immunity. Regarding the essential role played by glucose and insulin on immune cells, variations in their levels which occur in diabetes are most likely involved in immune disorders associated with this trait.

### 3.2. Induction of chronic diseases by microbiota dysbiosis

#### 3.2.1. Symbiosis between the host and its microbiota

As previously described (cf. Section 1.3), there is a permanent dialogue between the gastrointestinal tract and the intestinal microflora. The intestinal microbiota is a complex symbiotic ecosystem which has the capacity to (1) digest luminal component and (2) synthesize useful host nutrients, while (3) stimulating immune defense mechanisms. This symbiotic relationship between host and bacteria involves microbial fermentation processes. The predominant end-products of bacterial fermentation in the gut are short chain fatty acids, such as acetate, propionate, and butyrate. Acetate is taken up primarily by peripheral tissues and can also be utilized by adipocytes for lipogenesis [171]. The intestinal microflora also contributes to aminoacid synthesis. Indeed, high concentrations of urea are found in the colon of germ-free rats, indicating the role of bacteria in intestinal nitrogen recycling [172]. The intestinal ecosystem also plays a crucial role in the metabolism of lignan, a dietary phytoestrogen compound from plant origin, which could be involved in colon cancer, atherosclerosis, and diabetes. Moreover, it has been proposed that the microbiota deconjugates and dehydroxylates bile acids [173, 174], metabolizes bilirubin [175], reduces cholesterol [176], and degrades mucus glycoproteins produced by the intestinal epithelium’s goblet cell lineage [177].

As indicated above, the assembly of the gut microflora commences at birth and its composition will undergo dramatic changes during postnatal development. When space and nutrients are not limited, commensals with high division rates will predominate. As the population increases and nutrients are depleted, niches become occupied with more specialized species [178, 179]. The ability of other commensals to enter these occupied niches will depend on their ability to utilize the nutrients substrates more efficiently and/or to modify the nutrient reservoir to better suit their own metabolic needs. Therefore, an equilibrium between microbial nutrient utilization and host nutrient production should be achieved which is not deleterious for both partners [180].

Diet is clearly a key factor which regulates the sequence and the nature of colonization. In breast-fed infants, the intestinal flora is dominated by bifidobacteria, while formula-fed infants have a more diverse flora [180, 181]. In breast-fed infants, the microflora produces high amounts of acetate and lactate restricting the growth of potential pathogens such as *Escherichia coli* and *Clostridium perfringens* [182]. In comparison, formula-fed infants produce relatively high amounts of propionate and butyrate. The favored growth of bifidobacteria in breast-fed infants is likely due to the presence of neutral oligosaccharides with prebiotic effect, in the breast milk [183]. Similarly, it has been observed that the addition of prebiotics to infant regimen can stimulate growth of beneficial endogenous bacteria. As an example, feeding infants with formula enriched with galacto- and fructooligosaccharides significantly increased the number of bifidobacteria [184, 185].

#### 3.2.2. Alteration of the microbiota and outcome of chronic diseases

Although the composition of the microbiota varies along the length of the gut and during the life of the host, it is quite stable during a considerable part of a normal human lifespan. Recent metagenomic studies, however, showed that the microbial balance is altered in some immune disorders. Notably, a significant reduction in the diversity of the phyla Firmicutes has been reported in patients with Crohn’s disease (CD). While 43 distinct ribotypes of Firmicutes were identified in healthy microbiota, only 13 ribotypes were detected in CD patients, indicating a serious degree of microbial dysbiosis [186]. Moreover, new species have been identified in IBD patients, such as unclassified *Porphyromonadaceae* species. The authors suggested that the onset of the inflammatory disease could be due to this altered microbiota. Notably, loss of butyrate producers observed could upset the dialogue between host epithelial cells and resident microorganisms, hence contributing to the development of CD associated injury.

Given the worldwide epidemic in obesity, there is a growing interest concerning the interaction of the microbiota with the host in obese state. Previous experiments showed that colonization of the gut of germ-free mice with microbiota isolated from conventional animals led to a dramatic increase of 42% in body fat within 10–14 days, despite decreasing food consumption. Along the same lines, it was later shown that colonization of germ-free mice with an obese microbiota resulted in a significant greater increase in total body fat than colonization with a lean microbiota [187]. Altogether, these findings suggested that the microbiota of obese individuals may be more efficient at extracting nutritional value from a given diet than the microbiota of lean individuals [188, 189] and that this trait is transmissible by the microbiota. Furthermore, the comparison of the gut microbiota of leptin-deficient obese (*ob/ob*) mice versus lean mice showed that the relative abundance of the Bacteroidetesin *ob/ob* mice was 50%-lower, whereas that of the Firmicutes was 50%-higher [190].

Interestingly, similar results were reported in obese patients showing a decrease in the relative proportion of Bacteroidetes as compared to lean individuals [34]. Additionally, when obese patients lost weight over a one-year period, the proportion of Firmicutes became similar to that of lean individuals. Recently, the same authors showed that microbial colonization of gnotobiotic mice led to *de novo* lipogenesis and enhancement of adiposity associated with increased suppression of intestinal *Fiaf* expression, a circulating lipoprotein fasting inhibitor [191].

All these studies suggest that the obese state is associated with modifications in microbiota composition and that changes in microbial fermentation of dietary polyssacharides will influence intestinal absorption of monosaccharides and short-chain fatty acids and consequently their conversion to more complex lipids in the liver and deposit of lipids in adipocytes.

#### 3.2.3. Improvement of the beneficial effect of the microflora by probiotic supplementation

Individual human health is determined by a complex interplay between genes, environment, diet, lifestyle, and symbiotic gut microbial activity. Recognition of the interplay between genes and diet in the development of certain diseases and for maintenance of optimal metabolism has led to nutrigenomic or nutrigenetic approaches. These might allow to propose personalized or individualized nutrition in order to prevent, delay, and/or reduce the symptoms of some chronic diseases [192]. The ultimate goal of nutrigenomics is therefore to apply genomics, transcriptomics, proteomics, and metabolomics to human nutrition in order to get a better understanding of the relationship between health and nutrition. In addition, nutrigenomics will be useful to demonstrate the impact of bioactive food compounds on health and also the effect of healthy food on human health, therefore leading to the development of “functional food” which should keep individuals healthy according to their own needs.

The human microbiome project [193] aims to uncover the functional contributions of gut microbiota and to define how microbiota contributes to normal physiology and/or to predisposition to certain diseases. Nutrigenomics showed that diet can dramatically alter the microbial composition of gut microbiota. Current research increasingly recognizes the human gut microbiome as a metabolically versatile biological “digester” that plays an essential role in regulating the host metabolome [194]. Gut microbiota recovers energy and biologically active molecules from food which would otherwise be washed out by the intestinal tract without any benefit for the host. Indeed, predictions of microbial community metabolism, based on community gene content analysis, indicated that the obesity-associated gut microbiome has an increased capacity to harvest energy from the diet. Further, it is now clear that microbiota has profound regulatory effects outside the gut such as regulation of fat storage, maintenance of the intestinal barrier function, and modulation of the immune system. Dysbiosis has been reported both in obesity and chronic inflammatory bowel diseases and a deficiency in “good bugs” such as lactobacilli and bifidobacteria has been observed in individuals having a western type of lifestyle.

The demonstration of the importance of human gut microbiota in health restoration and maintenance has kindled an interest in probiotics, defined as microbial food supplements which beneficially affect the host by improving its intestinal microbial balance. It is now well accepted that supplemented probiotic bacteria might have the capacity to improve the functions of both the innate immune system and the gut physiology. Indeed, regular intake of probiotic bacteria has been shown to maintain the gut immune homeostasis by altering microbial balance or by interacting with the gut immune system, explaining their potential effect in gastrointestinal diseases. Probiotics have proven benefits in treatment or prevention of certain type of diarrhea [195], inflammatory bowel diseases [196, 197], some cancers [198], and food allergy and atopic eczema in children [199]. Although there is now considerable body of information concerning the clinical efficiency of probiotics, their mechanisms of action remain unclear. Their beneficial effects can be exerted through different means, such as production of antimicrobial metabolites, competitive exclusion of enteric pathogens, or neutralization of dietary carcinogens. Their capacity to modulate the mucosal immune system is regarded as one of the most obvious beneficial properties. Indeed we, and others, showed that probiotics present distinct strain-specific immunomodulatory capacities in vitro [200] which can be closely correlated with their in vivo anti-inflammatory potential [201]. We also reported the importance of cell wall components in the pro- versus anti-inflammatory properties of lactobacilli [202]. Interestingly, the anti-inflammatory effects of lactobacilli observed after either oral or systemic administration [203] suggest that the protective mechanisms might involve regulatory cell populations. Recent studies reported that a defined probiotic mixture ameliorates murine colitis by inducing regulatory T cells [204] and can induce in vivo peripheral T cell hyporesponsiveness [205], suggesting a modulation through dendritic cell (DC) function. We recently showed that selected strains indeed are able to induce tolerogenic dendritic cells that confer protection in a murine model of colitis upon adoptive transfer. This capacity was dependant on both TLR2 and NOD2 signalings, confirming a key role of cell wall structures [206].

Regarding obesity, only few studies have addressed the potential effects of probiotics in the management of this disease. Since obesity is presently viewed as an inflammatory disease, affecting both innate and acquired immune systems [156], we could speculate that probiotics with potential anti-inflammatory properties could counteract the development of complications associated with this pathology. Recently, Bleau et al. [207] reported that supernatants from lactobacilli-treated adipocytes decreased the inflammatory-type response of lymphocytes. These effects were correlated with a reduction of leptin production by lactobacilli-treated adipocytes. Finally, a selected strain of *Lactobacillus rhamnosus* has been reported to protect mice from diet-induced obesity, likely due to the production of conjugated linoleic acid by the bacteria [208].

From the limited, yet convincing, studies performed so far, one can predict that nutrigenomics will improve our knowledge on the function of gut microbiota and allow therapeutic manipulation of the gut ecosystem to become a valid and realistic future prospect.

## 4. CONCLUSIVE REMARKS

The rapid rise in the numbers of obese patients, partly due to a lifestyle that promotes overeating and inactivity, is presently a critically important health issue worldwide. Obesity is associated with a number of diseases collectively summarized as the “metabolic syndrome,” involving insulin resistance, type 2 diabetes, and cardiovascular diseases.

Although obesity results from complex and multiple interactions between genetic and environmental factors, numerous studies provide strong corroborative evidences that overnutrition can promote metabolic diseases.

Like other chronic disorders of metabolic homeostasis, we showed that obesity is also associated with immune disbalances, involving low but chronic level of inflammation, as well as infiltration of adipose tissue with activated macrophages.

It has been proposed that chronic activation of the innate immune system could be regarded as a possible risk factor in the development of obesity and its associated inflammation. Indeed, signaling receptors of the innate immune system (such as TLRs) induce signal transduction pathways that lead to the activation of transcription factors which are also activated in response to proinflammatory cytokines and which ultimately suppress the insulin signaling pathway. Therefore innate immunity, in addition to its immediate response to pathogens, may also be involved in whole-body and organ-specific insulin sensitivity as well as in the regulation of the energy balance. Interestingly TLRs, notably TLR4, expressed on both innate and adaptive immune cells, are also found on cells of insulin-responsive tissue such as adipocytes. TLRs may therefore represent a potential molecular gate linking inflammation with insulin resistance, diabetes, and obesity.

The second aspect developed in this review concerns the critical importance of the gut microbiota in the development of metabolic diseases, particularly obesity. As described, this hypothesis started with the fascinating observation that young adult germ-free mice had only half of the body fat of their conventional counterparts receiving the same diet. We attempted to compile the numerous benefits that arise from a healthy intestinal microbiota (extraction of nutriments from food; participation in the development and maturation of the gut immune system, and regulation of fat storage within adipocytes) and discussed the potential role of a disturbed flora in metabolic disorders such as obesity. Again, TLRs appeared to be the link between nutrition, microbiota, and inflammation.

**Figure 1:**
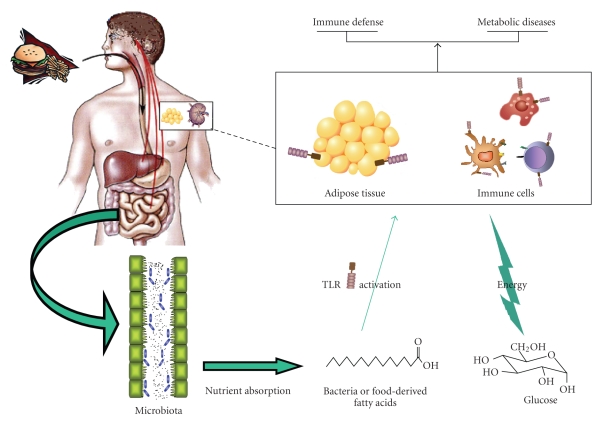
After a meal, fatty acids and glucose, through intestinal absorption, enter the blood. Both serve as fuels for cells or tissues, glucose being the most important to fulfill the energy requirement of immune cells, and lipids representing major components of cell membranes. Besides, food-derived fatty acids, as well as intestinal bacteria-derived fatty acids could be sensed by Toll-like receptors (TLRs) which are expressed on immune cells, adipocytes or intestinal gut, resulting in activation of the immune system. Depending on the intensity, the time lasting, and the control of these events, it will either favor the development of an efficient immune defense, or lead to a drift towards metabolic diseases such as obesity.

Finally, we showed that immune cells, both from the innate and adaptive immune systems, express TLRs and that immune responses depend on a critical increase in energy requirements, preferably met by glucose. Such observations allow to deduce a quasi parallel between lymphocyte glucose metabolism and bodily metabolism mostly via the insulin signaling pathway, and reinforce the link between nutrition, immune system, energy metabolism, and gut microbiota, resumed in Figure 1.
